# Right Ventricular Strain in Healthy Children: Insights from Speckle-Tracking Echocardiography

**DOI:** 10.3390/jcdd12090322

**Published:** 2025-08-22

**Authors:** Renée S. Joosen, Eva A. M. Meulblok, Esther H. Mauritz-Fuite, Martijn G. Slieker, Johannes M. P. J. Breur

**Affiliations:** Department of Pediatric Cardiology, University Medical Center Utrecht, Lundlaan 6, 3584 EA Utrecht, The Netherlands

**Keywords:** pediatric strain, speckle-tracking echocardiography, normal values, right ventricle, left ventricle

## Abstract

Background: Right ventricular (RV) strain using two-dimensional speckle tracking is a reliable and clinically significant tool for detecting RV systolic dysfunction, but it varies by age, vendor, and software. Objectives: To establish pediatric age-specific normal values and Z-score equations for biventricular strain using GE Healthcare equipment and software. Methods: Children 0–18 years with structurally and functionally normal hearts, who visited the Wilhelmina Children’s Hospital Utrecht between October 2020 and December 2023, were retrospectively included and divided into age groups: 0 years, 1–4 years, 5–9 years, 10–13 years, and 14–18 years. Left ventricular (LV) and RV global longitudinal strain (GLS) and RV free wall longitudinal strain (FWGLS) were analyzed. Results: We included 129 subjects (57% male) (0 years: n = 17; 1–4 years: n = 22; 5–9 years: n = 34; 10–13 years: n = 35; 14–18 years: n = 20). Low R^2^ values were strain-adjusted for age, height, and body surface area (all < 0.3), and the sample size limited Z-score equation reliability. Therefore, data are presented as mean ± SD or median [IQR] stratified by age. LV GLS, RV GLS, and RV FWGLS showed a nonlinear relationship with age, peaking at the 1–4 years age group and decreasing with age. Conclusions: LV GLS, RV GLS, and RV FWGLS showed age-related differences in children using GE equipment and software, which highlights the importance of age-specific normal strain values, including Z-score equations as a function of age.

## 1. Introduction

Right ventricular (RV) systolic function is an independent predictor of a poor outcome, significantly impacting the prognosis of patients with congenital heart diseases [1,2,3]. While RV ejection fraction (RVEF) measured by Cardiac Magnetic Resonance Imaging (CMR) is the gold standard for assessing RV function, its use is limited by availability, lengthy acquisition times, movement artefacts, and contraindications such as the presence of medical devices [4]. Echocardiography, a more accessible and commonly used alternative, traditionally relied on RV parameters that were load and angle-dependent [5]. However, the relatively new technique of speckle tracking measures RV strain, representing myocardial deformation, and offers the benefit of being less influenced by load compared to traditional RV function indices, and is less affected by RV geometry [5,6,7,8]. Previous studies have demonstrated that RV strain is a reliable and clinically significant tool for detecting RV systolic dysfunction and may even be more sensitive than RVEF [9,10]. However, strain measurements can vary based on age, vendor, or software, and the primary obstacle to incorporating RV strain into pediatric clinical practice is the absence of Z-score equations [11,12,13]. Therefore, this study aims to establish pediatric age-specific normal values and Z-score equations for biventricular strain using two-dimensional speckle tracking with GE Healthcare equipment and software.

## 2. Methods

### 2.1. Study Population

This retrospective study was conducted at the Wilhelmina Children’s Hospital in Utrecht, The Netherlands. Echocardiograms of children aged 0–18 years old visiting the outpatient clinic were retrospectively obtained between October 2020 and December 2023. Children were included if they had a morphologically and functionally normal heart with no history or suspicion of cardiovascular or lung disease. They were excluded if they had an underlying oncological disease, pathological physical examination, a history of familial cardiomyopathies, genetics associated with cardiomyopathies, hypertension, or abnormal 12-lead electrocardiographic findings (Supplementary Figure S1). Indications for echocardiography included innocent cardiac murmurs, chest pain, dizziness or collapse complaints, fatigue, or a familial cardiac screening for congenital heart disease or a bicuspid aortic valve (Supplementary Figure S2). The subjects were divided into age groups: 0 years, 1–4 years, 5–9 years, 10–13 years, and 14–18 years. This study was approved by the Institutional Ethics Committee of the University Medical Center Utrecht, and due to the extensive design of this study, the right of no objection was used (24U-0042).

### 2.2. Image Acquisition

All echocardiograms were acquired using GE Healthcare E95 echocardiography machines (GE Healthcare, Chicago, IL, USA) according to a standardized protocol described before [14]. A frame rate of at least 50 frames/s was accepted for deformation imaging [15]. In accordance with the 2024 American Society of Echocardiography guidelines for performing a comprehensive pediatric TTE, a RV-focused apical four-chamber (A4CH) view was used to obtain RV strain to ensure full visualization of the RV chamber throughout the cardiac cycle [16,17,18,19]. LV strain was obtained using the apical three-chamber (A3CH) view, apical two-chamber (A2CH), and A4CH view (Supplementary Figure S3), [18].

### 2.3. Post-Processing

Post-processing of RV and left ventricular (LV) longitudinal strain was performed by an experienced observer using the Q-analysis application from EchoPAC software (GE Healthcare, Chicago, IL, USA, version 203) as described before [14]. Software for LV strain analysis was applied to the RV. The endocardial borders were traced manually in end-systolic frames, with adjustments made to the region of interest (ROI) if necessary. End-systole was determined at the closure of the aortic valve for both LV and RV [20]. If there was a significant difference in the R-R interval between different views, valve closure was assessed visually. RV global longitudinal strain (GLS) was defined as the average peak strain of the entire RV lateral wall and septal wall, while RV free wall longitudinal strain (FWGLS) was defined as the average peak strain of the three segments of the RV lateral wall. LV peak strain values from A2CH, A3CH, and A4CH views were averaged to obtain LV GLS. Segments were excluded in case of sub-optimal tracking of the ROI with no improvements after manual adjustments. A shift towards more negative strain values (e.g., from −19% to −21%) was defined as an increase in strain or higher strain, while a shift towards more positive strain values (e.g., from −21% to −19%) was defined as a decrease in strain or lower strain. Additional parameters obtained included strain rate, RV fractional area change (FAC), tricuspid annular plane systolic excursion (TAPSE), and LV ejection fraction (LVEF) using the biplane method.

### 2.4. Statistical Analysis

Statistical analysis was conducted using IBM SPSS Statistics (version 29.0), and figures were generated with Graphpad Prism (version 10.2.0). Continuous variables were tested for normality using the Shapiro-Wilk test and presented as mean ± standard deviation (SD) or median [interquartile range (IQR)]. Categorical variables were displayed as frequencies (percentages). Differences in continuous and categorical variables were assessed using an independent samples *t*-test, chi-square test, and Fisher’s exact test. Differences between age groups were analyzed using one-way analysis of variance (ANOVA) with Bonferroni post hoc analysis for normally distributed continuous outcomes; for non-normally distributed variables, the Kruskal-Wallis test with Mann-Whitney U post hoc test was applied. If the Levene’s test for homogeneity of variances was not met for ANOVA, a Welch ANOVA with Games-Howell post hoc analysis was used. Results were deemed statistically significant with a two-tailed *p*-value < 0.05. Several models (linear, logarithmic, quadratic, and cubic) were considered for creating Z-score equations, defining Z as the number of SDs greater than or less than the predicted mean strain value. Strain values were modeled with age, height, and body surface area (BSA), calculated using the Dubois formula. Goodness of fit (R^2^) was assessed to determine the best-fitting model.

## 3. Results

### 3.1. Study Population

A total of 888 subjects were initially deemed eligible for the study. Of these, 706 were excluded due to the absence of an RV-focused 4ACH view for RV strain analysis. This left 182 subjects, but 53 were further excluded due to poor echocardiographic quality. As a result, 129 subjects (57% male, age range: 32 days–18 years) were included and categorized into the following age groups: 0 years (n = 17), 1–4 years (n = 22), 5–9 years (n = 34), 10–13 years (n = 35), and 14–18 years (n = 20) (Supplementary Figure S1). The most common indications for echocardiography were cardiac screening and cardiac murmur, with older age groups more frequently presenting indications such as chest pain, dizziness, palpitations, and collapse complaints (Supplementary Figure S2). Patient characteristics and conventional echocardiographic parameters are summarized in Table 1.

### 3.2. Normal Values RV Strain

**Figure 1:**
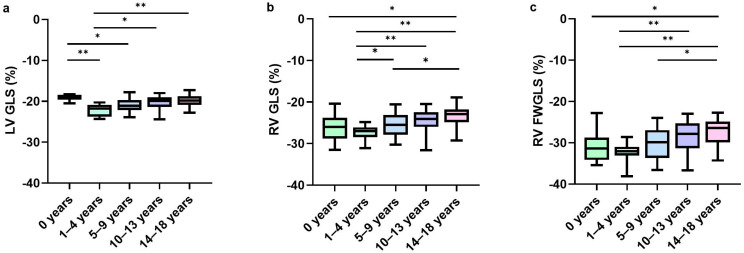
**Normal RV and LV strain by age group.** * *p* < 0.05; ** *p* < 0.001. Graphs show normal values of LV global longitudinal strain (**a**), RV global longitudinal strain (**b**), and RV free wall global longitudinal strain (**c**) by age group. FWGLS = free wall longitudinal strain; GLS = global longitudinal strain; LV = left ventricle; RV = right ventricle.

RV strain values among the different age groups are displayed in Table 2. RV GLS, RV FWGLS strain, and strain rates varied by age group. There was a nonlinear relationship between RV strain values and age. For both RV GLS and RV FWGLS, strain values increased from the age group of 0 years to 1–4 years, reaching the most negative values in the 1–4 years age group. Subsequently, RV strain decreased with age in the older age groups (Central Illustration, Figure 1). Mean strain rates of RV GLS and RV FWGLS decreased across the age groups. RV FWGLS resulted in more negative strain values compared to RV GLS. RV GLS and RV FWGLS showed moderate correlations with LV GLS (R = 0.450, *p* < 0.001; R = 0.353, *p* = 0.002, respectively). No correlation was observed between RV GLS or RV FWGLS and RV FAC or TAPSE.

In the overall cohort, RV FWGLS was significantly higher in females compared to males (females: −30 ± 4%, males: −29 ± 4%, *p* = 0.047), while no difference was observed in RV GLS. Mean RV GLS and RV FWGLS, stratified by age group and gender, are detailed in Supplementary Table S1. In the 5–9 years age group, RV FWGLS was significantly higher in females compared to males (−31.6 ± 3.5% vs. −28.7 ± 3.8%, *p* = 0.028). Additionally, in the oldest age group (14–18 years), both RV GLS were significantly higher in females than in males (RV GLS: 24.1 ± 2.1% vs. 21.9 ± 2.1%, *p* = 0.042). Significant differences in RV GLS and RV FWGLS were observed across age groups in males only, with the most pronounced differences occurring between the 1–4 years group and the older age groups (5–9 years, 10–13 years, and 14–18 years) (Supplementary Table S1).

### 3.3. Normal Values LV Strain

Normal values for LV GLS by age group are presented in Table 2. Mean LV GLS and strain rate exhibited variation across age groups, displaying a nonlinear relationship with age. The pattern in LV strain mirrored that of RV strain, with values becoming more negative with age, peaking in the 1–4 years age group, and subsequently declining with further aging (Central Illustration, Figure 1). LV GLS showed a poor correlation with LVEF (R = −0.288; *p* = 0.028). No significant differences in LV GLS were found between males and females, either in the overall cohort or across individual age groups (Supplementary Table S1).

### 3.4. Z-Score Equations

In all regression analyses, low coefficients of determination (R^2^) were found among all strain parameters (LV GLS, RV GLS, and RV FWGLS) in association with age, height, and BSA (all R^2^ < 0.3). Additionally, small sample sizes were available per age group. These factors hindered the development of sufficiently reliable Z-score equations. Therefore, data were solely presented as mean ± SD or median [IQR] stratified by age group and divided by gender.

## 4. Discussion

To the best of our knowledge, this study is the largest to establish normal values for RV strain in children aged 0–18 years, utilizing GE Healthcare equipment and software. The primary findings indicate that RV GLS, RV FWGLS, and LV GLS vary with age: increasing after birth, peaking in the 1–4 year age group, and then gradually decreasing as age advances. The low coefficients of determination (R^2^) and the small sample size limited the ability to develop Z-score equations, resulting in the data being presented as mean ± SD or median [IQR]. The high exclusion rate due to missing RV-focused A4CH views or suboptimal image quality highlights a significant barrier to reliable RV strain measurement in children. This underscores the need for standardized acquisition protocols. We therefore recommend incorporating dedicated RV-focused A4CH into routine pediatric echocardiography, an approach also endorsed by international guidelines, and enhancing sonographer training to improve the feasibility and accuracy of RV strain analysis.

This study provides normal values for biventricular strain in a relatively large cohort of healthy children across all pediatric age groups, utilizing vendor-specific software (GE Healthcare, Chicago, IL, USA). Although research on normal values and Z-score equations for LV GLS in children is relatively common, studies focusing on RV strain are less frequent. Recently, Romanowicz et al. evaluated normal values and Z-score equations for both RV and LV strain in a cohort of over 1000 children using the Philips EPIQ platform and QLAB software (QLAB 10.8 vs. AutoSTRAIN) [21]. However, studies evaluating RV strain using GE Healthcare software have generally involved small sample sizes, with all studies including fewer than 50 subjects [22]. Levy et al. conducted a meta-analysis of studies using GE Healthcare software, which resulted in a larger sample size. However, this analysis pooled data from control groups and lacked the ability to stratify by age groups, unlike a study specifically designed to provide normative data [22].

We observed that strain values vary by age group in a nonlinear fashion, which supports previous findings [21,23,24,25,26,27]. Some studies have not found differences in RV strain across age groups, which may be attributed to variations in vendors, sample sizes, or differences in age group distributions [23,28]. In our study, RV GLS, RV FWGLS, and LV GLS increased after birth, reaching a maximum in the age group of 1–4 years and subsequently decreased with age. This pattern has previously been reported by Romanowicz et al., and a similar trend is observed in a few other studies [21,27,29,30,31]. Although RV and LV function were within normal limits based on conventional echocardiographic parameters and strain, we observed little to no correlations. This may be partly due to the limited sample size, but also reflects fundamental differences in what these measures assess. TAPSE, for example, reflects only basal longitudinal motion, while RV strain captures global myocardial deformation and is less angle-dependent. Larger, well-powered studies are needed to more accurately determine the relationship between strain and conventional echocardiographic parameters in healthy children.

Notably, RV strain has been shown to change throughout maturation from infancy to adolescence [28,32]. In fetal circulation, the RV functions as the systemic pump, matching the LV in both pressure and wall thickness. After birth, a significant drop in pulmonary vascular resistance decreases RV afterload and pulmonary artery (PA) pressure, leading to increased RV deformation [30,33]. RV contractility may temporarily decrease but recovers through ventriculo-arterial coupling mechanisms [31]. Studies have shown that RV GLS and RV FWGLS increase from the first week after birth to approximately one year of age due to neonatal load changes and enhanced contractility [31,34,35,36]. However, the question remains whether this reflects a genuine increase in contractility or is the result of cardiac myocyte rearrangement and reduced RV afterload [36]. After toddler age, RV strain appears to decline with age, but this is unlikely due to increased afterload or reduced contractility. Recent studies indicate that pulmonary artery pressure and pulmonary vascular resistance both decrease with age [37,38]. Furthermore, Colan et al. demonstrated minimal changes in RV contractility after the age of 4 years [39]. Instead, this decline may be linked to maturational differences in directional contraction. Research indicates that in older children, global RV function mainly depends on anteroposterior contraction, whereas in toddlers, both longitudinal and anteroposterior contractions equally contribute to RV function [29]. During anteroposterior contraction, RV free wall insertion lines move toward each other due to LV circumferential shortening, likely driven by shared LV and RV myocardial fibers and the interventricular septum [29,40]. The concept of interventricular interdependence is further supported by a correlation between RV and LV strain, with previous studies indicating that 20–40% of RV pressure increase is directly related to LV contraction [41]. RV FWGLS was higher than RV GLS, presumably because the interventricular septum is part of the LV, which primarily relies on circumferential rather than longitudinal contraction. This finding is consistent with other literature [11,24,28,32,42]. We found significantly higher RV FWGLS for females compared to males across the entire group. Sex differences in RV strain may be influenced by various factors such as hormonal, physiological, or maturational changes. While investigating these contributors was beyond the scope of the current study, they represent an important area for future research, as they could offer valuable insights into sex-specific patterns in normal RV maturation. Additionally, and in line with other studies, we did observe more negative RV strain values in the oldest age group, potentially related to puberty [21,23,43].

### 4.1. Limitations

This study has several limitations that are inherent to the study design. Firstly, 80% of eligible patients were excluded due to the absence of an RV-specific A4CH, as this is not part of the standardized echo protocol in our center. A dedicated RV A4CH is essential for reliable RV strain analysis because a standard A4CH does not fully capture the RV free wall, which lies outside the primary imaging plane due to the RV’s complex geometry and its position behind the sternum, further limiting visualization. Incomplete visualization can lead to underestimation or variability in strain measurements and supports standardization of a dedicated RV A4CH in pediatric echocardiography. Additionally, approximately one-third of the patients were excluded due to the poor quality of echocardiography, making RV strain analysis infeasible. This underscores the importance of specialized sonographer training to consistently obtain the specific images required for accurate RV strain analysis. Not all LV images were obtained from every subject, which may have affected LV GLS measurements. Additionally, race and ethnicity were not considered, and RV strain was derived from a single view, assessing only RV inflow and potentially not offering a comprehensive evaluation of global RV function [14]. Grouping of all infants under the 0-year age category, which encompasses a highly dynamic physiological period (1 to 12 months), marked by rapid changes in pulmonary vascular resistance, RV loading conditions, and myocardial structure. Although the sample size did not allow for further stratification, future studies should consider subdividing this age group (e.g., neonates, 1–6 months, 6–12 months) to better capture early postnatal myocardial adaptation. Moreover, the low correlation between strain and age, height, and BSA, indicated by the coefficient of determination (R^2^), and the sample size hampered the creation of reliable Z-score equations. In order to do so, an R^2^ value greater than 0.6 and a sample size of 80 subjects per age group, ideally 140 subjects, is recommended [23,44,45]. Due to these unmet requirements, we chose not to provide Z-score equations to avoid potentially misleading clinical results. Instead, data were presented as mean ± SD or median [IQR], stratified by age group, as previously done [23,46].

### 4.2. Clinical Implications

The use of RV strain in echocardiography shifts the focus from qualitative observation of RV function to quantitative assessment. Establishing age-related and vendor-specific normal values for RV strain enhances our understanding of normal maturational changes in pediatric ventricular function, potentially increasing sensitivity for detecting cardiac diseases in this population. However, a key barrier to the clinical implementation of RV strain measurements is the lack of Z-score equations. In the current study population, the development of reliable Z-score equations was not feasible, and they were therefore not reported, as this could lead to unreliable values and potential clinical misclassification. Future research should focus on larger studies to reliably develop Z-score equations as a function of age for GE software and equipment, facilitating the integration of RV strain into routine pediatric clinical practice. Additionally, consistent with current guidelines, it is recommended that a standardized, RV-focused A4CH view will be included in pediatric echocardiography protocols to ensure accurate and reproducible RV strain measurements.

## 5. Conclusions

LV GLS, RV GLS, and RV FWGLS showed age-related differences in children using GE equipment and software, which highlights the importance of age-specific normal strain values, including Z-score equations as a function of age.

## Figures and Tables

**Table 1:** Baseline characteristics.

	0 Years (n = 17)	1–4 Years (n = 22)	5–9 Years (n = 34)	10–13 Years (n = 35)	14–18 Years (n = 20)	*p*-Value
**Male, n (%)**	8 (47)	14 (64)	18 (53)	23 (66)	10 (50)	*p* = 0.593
**Height (cm), mean ± SD**	63 ± 6	98 ± 8	126 ± 12	156 ± 13	173 ± 11	*p* < 0.001
**Weight (kg), median [IQR)**	6 (5–8)	16 (14–18)	25 (20–31)	42 (34–49)	61 (55–73)	*p* < 0.001
**Body mass index (kg/m^2^), median [IQR]**	16 (15–17)	16 (16–17)	15 (14–17)	18 (16–19)	20 (19–23)	*p* < 0.001
**Body surface area (m^2^), mean ± SD**	0.32 ± 0.06	0.64 ± 0.09	0.95 ± 0.17	1.39 ± 0.21	1.76 ± 0.18	*p* < 0.001
**Heart rate (bpm), median [IQR]**	138 (131–150)	94 (85–109)	80 (73–99)	73 (65–83)	77 (58–94)	*p* < 0.001
**LVEF (%), median [IQR]**	54 (53–54)	58 (55–61)	57 (53–60)	57 (56–59)	59 (55–60)	*p* = 0.758
**RV FAC (%), mean ± SD**	50 ± 7	45 ± 6	44 ± 7	46 ± 5	45 ± 5	*p* = 0.014
**TAPSE (mm), mean ± SD**	14.7 ± 3.2	19.8 ± 2.0	20.8 ± 2.1	22.2 ± 2.4	22.1 ± 1.9	*p* < 0.001

FAC = fractional area change; LVEF = left ventricular ejection fraction; RV = right ventricle; TAPSE = tricuspid annular plane systolic excursion.

**Table 2:** Normal strain values by age group.

	0 Years (n = 17) (1)	1–4 Years (n = 22) (2)	5–9 Years (n = 34) (3)	10–13 Years (n = 35) (4)	14–18 Years (n = 20) (5)	*p*-Value	Post Hoc
**Right ventricle**							
FR (fr/s), median [IQR]	77 (72–89)	70 (61–74)	65 (60–76)	65 (60–75)	70 (60–91)	*p* = 0.061	-
RV GLS (%), mean ± SD	−26.2 ± 3.3	−27.2 ± 1.5	−25.5 ± 2.7	−24.4 ± 2.8	−23.1 ± 2.3	*p* < 0.001	1 vs. 5; 2 vs. 3, 4 and 5; 3 vs. 5
SR RV GLS (1/s), median [IQR]	−2.4 (−3.1–−2.1)	−2.1 (−2.2–−1.7)	−1.6 (−1.9–−1.5)	−1.8 (−2.0–−1.5)	−1.6 (−1.8–−1.5)	*p* < 0.001	1 vs. 2, 3, 4 and 5; 2 vs. 3, 4 and 5
RV FWGLS (%), mean ± SD	−30.9 ± 3.4	−32.4 ± 2.1	−30.0 ± 3.9	−28.4 ± 3.7	−27.0 ± 3.2	*p* < 0.001	1 vs. 5; 2 vs. 4 and 5; 3 vs. 5
SR RV FWGLS (1/s), median [IQR]	−3.0 (−3.5–−2.6)	−2.4 (−2.7–−2.0)	−1.9 (−2.3–−1.6)	−1.8 (−2.0–−1.5)	−1.6 (−1.8–−1.5)	*p* < 0.001	1 vs. 3, 4 and 5; 2 vs. 3, 4 and 5
**Left ventricle**							
FR (fr/s), median [IQR]	85 (80–91)	63 (58–82)	65 (59–66)	61 (55–77)	61 (56–89)	*p* = 0.066	1 vs. 4
LV GLS (%), median [IQR]	−19.2 (−19.2–−18.4)	−21.8 (−23.7–−20.9)	−21.1 (−22.1–−19.7)	−20.3 (−21.4–−19.2)	−20.4 (−21.0–−18.8)	*p* = 0.003	1 vs. 2
LV GLS A2CH view (%), mean ± SD	−19.5 ± 1.4	−22.0 ± 1.2	−21.4 ± 2.5	−21.2 ± 1.6	−21.1 ± 1.4	*p* = 0.358	-
LV GLS A3CH view (%), mean ± SD	−19.0 ± 0.9	−21.6 ± 2.0	−20.7 ± 1.6	−20.4 ± 2.3	−20.1 ± 1.6	*p* = 0.065	-
LV GLS A4CH view (%), mean ± SD	−18.8 ± 1.7	−22.1 ± 1.7	−20.3 ± 1.9	−19.8 ± 1.8	−19.6 ± 1.4	*p* = 0.002	1 vs. 2; 2 vs. 4 and 5
SR LV GLS (1/s), median [IQR]	−1.7 (−1.9–−1.6)	−1.4 (−1.5–−1.3)	−1.4 (−1.5–−1.3)	−1.2 (−1.3–−1.1)	−1.2 (−1.4–−1.1)	*p* < 0.001	1 vs. 4 and 5; 3 vs. 4

A2CH = apical two-chamber view; A3CH = apical three-chamber view; A4CH = apical four-chamber view; FR: frame rate, FWGLS: free wall global longitudinal strain, GLS: global longitudinal strain, LV = left ventricle; RV = right ventricle; SR: strain rate.

## Data Availability

Due to the nature of the data and the right of no objection, the anonymous dataset will only be available after a granted collaboration to the corresponding author.

## Supplementary Materials

The following supporting information can be downloaded at: https://www.mdpi.com/article/10.3390/jcdd12090322/s1, Figure S1: Flowchart included subjects; Figure S2: Indications for echocardiography; Figure S3: Echocardiographic views used to assess RV strain and LV global longitudinal strain; Table S1: Normal strain values by age group divided for gender.
