# Supplementary material for: Right Ventricular Strain in Healthy Children: Insights from Speckle-Tracking Echocardiography
Source: J Cardiovasc Dev Dis. 2025 Aug 22;12(9):322. doi: 10.3390/jcdd12090322 (PMC12470735; doi:10.3390/jcdd12090322)
Supplement: Supplementary file 1 [file jcdd-12-00322-s001.zip › Supplemental Material.pdf]

Supplemental material

Supplemental Figure S1. Flowchart included subjects

RV= right ventricle

Supplemental Figure S2. Indications for echocardiography

Supplemental Figure S3. Echocardiographic views used to assess RV strain and LV global longitudinal strain.

Right ventricular global longitudinal strain (RV GLS) was defined as the average peak strain of the entire RV lateral wall and septal wall. Right ventricular free wall longitudinal strain (RV FWGLS) was defined as the average peak strain of the three segments of the RV lateral wall. Left ventricular global longitudinal strain (LV GLS) was obtained by averaging the peak strain values from the apical two-chamber (A2CH), three-chamber (A3CH), and four-chamber (A4CH) views.

Supplemental Table S1. Normal strain values by age group divided for gender

	0 years (n=17) (1)	1-4 years (n=22) (2)	5-9 years (n=34) (3)	10-13 years (n=35) (4)	14-18 years (n=20) (5)	p-value	Post hoc
**Males**							
LV GLS (%), median [IQR]	-19.4 [-19.6 – -19.4]	-21.8 [-23.6 – -20.9]	-20.5 [-20.5 – -19.1]	-20.7 [-22.3 – -19.2]	-19.2 [-21.2 – -18.8]	p=0.078	-
RV GLS (%), mean ± SD	-26.6 ± 4.1	-27.1 ± 0.9	-24.8 ± 2.8	-24.5 ± 3.2	-21.9 ± 2.1*	p<0.001	2 vs. 3, 4 and 5; 3 vs. 5
RV FWGLS (%), mean ± SD	-30.6 ± 4.3	-32.3 ± 1.2	-28.7 ± 3.8*	-28.2 ± 4.0	-25.5 ± 2.6	p<0.001	2 vs. 3, 4 and 5
**Females**							
LV GLS (%), median [IQR]	-18.8 [-20.1 – -18.3]	-21.7 [-23.8 – -20.8]	-21.4 [-22.1 – -19.7]	-19.9 [-20.6 – -19.1]	-20.7 [-21.2 – -19.9]	p=0.031	-

RV GLS (%), mean ± SD	-25.7 ± 2.7	-27.2 ± 2.2	-26.3 ± 2.4	-24.2 ± 2.0	-24.1 ± 2.1*	P=0.017	-
RV FWGLS (%), mean ± SD	-31.2 ± 2.6	-32.5 ± 3.2	-31.6 ± 3.5*	-28.8 ± 3.4	-28.3 ± 3.2	P=0.021	-

*p<0.05 between males and females. FWGLS: free wall global longitudinal strain, GLS: global longitudinal strain, LV= left ventricle; RV= right ventricle
