# Supplementary figures and images for: Right Ventricular Strain in Healthy Children: Insights from Speckle-Tracking Echocardiography
Source: J Cardiovasc Dev Dis. 2025 Aug 22;12(9):322. doi: 10.3390/jcdd12090322 (PMC12470735; doi:10.3390/jcdd12090322)

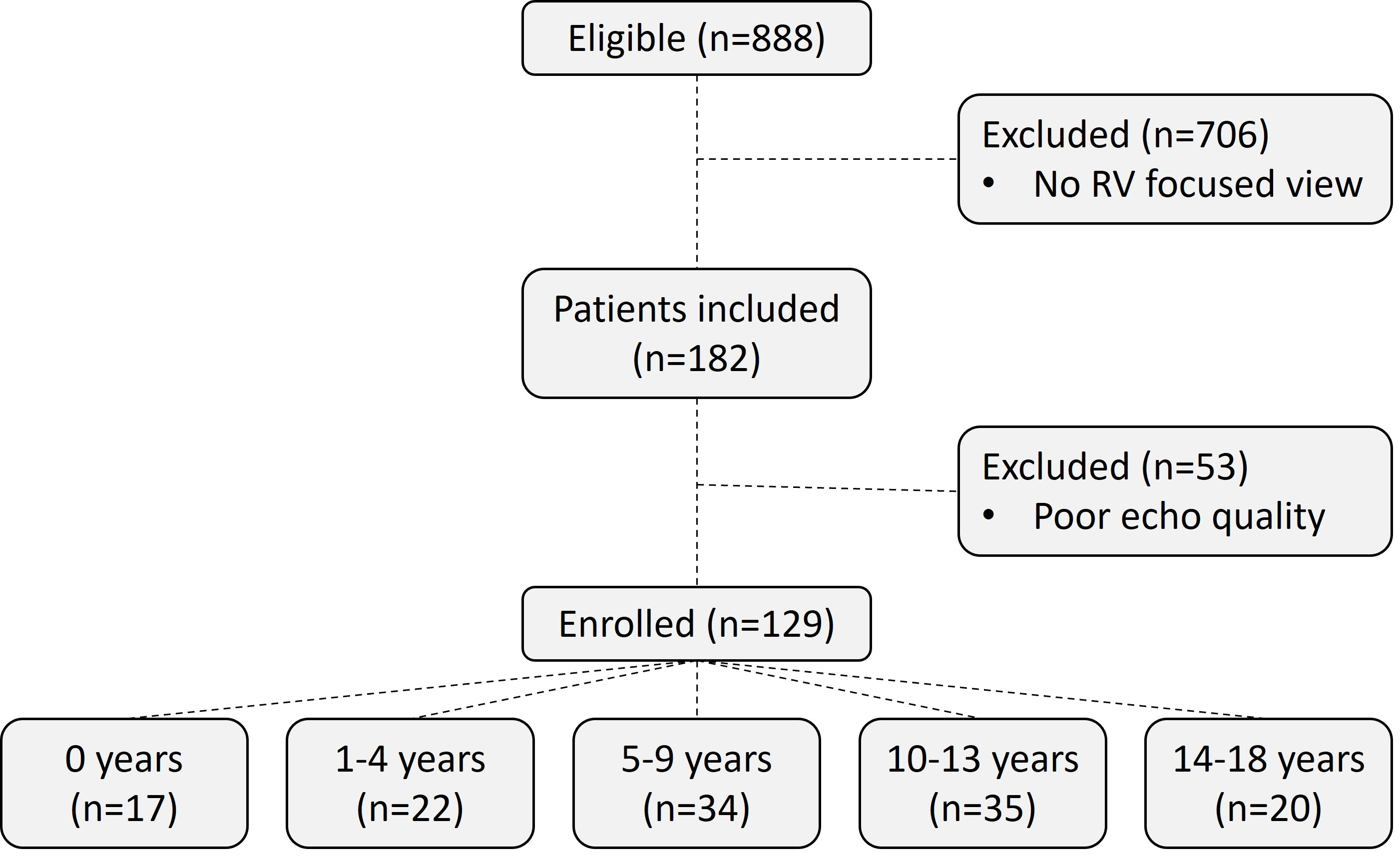

Supplement: Supplementary file 1 [file jcdd-12-00322-s001.zip › Supplemental Figure S1.tif]

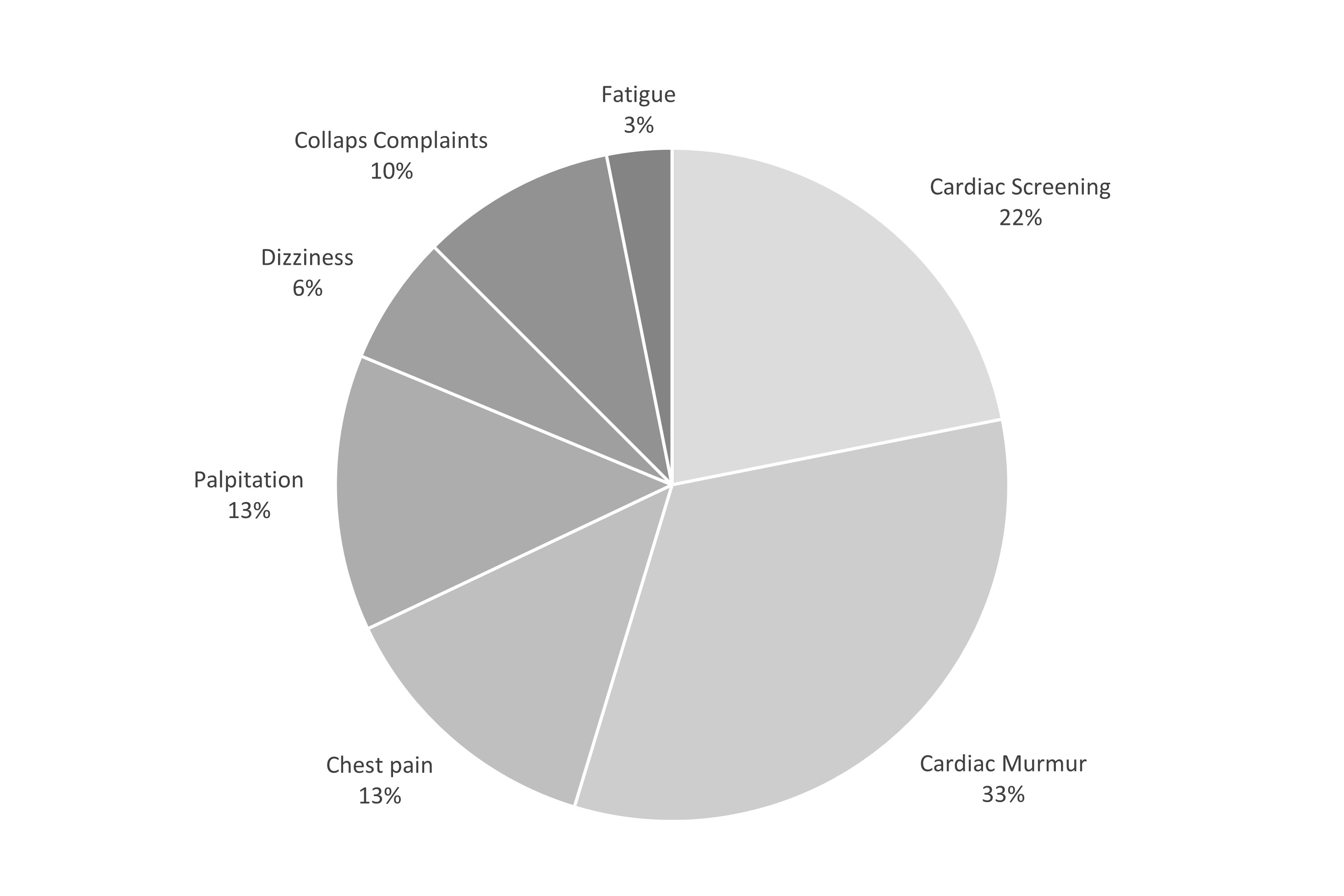

Supplement: Supplementary file 1 [file jcdd-12-00322-s001.zip › Supplemental Figure S2.tif]

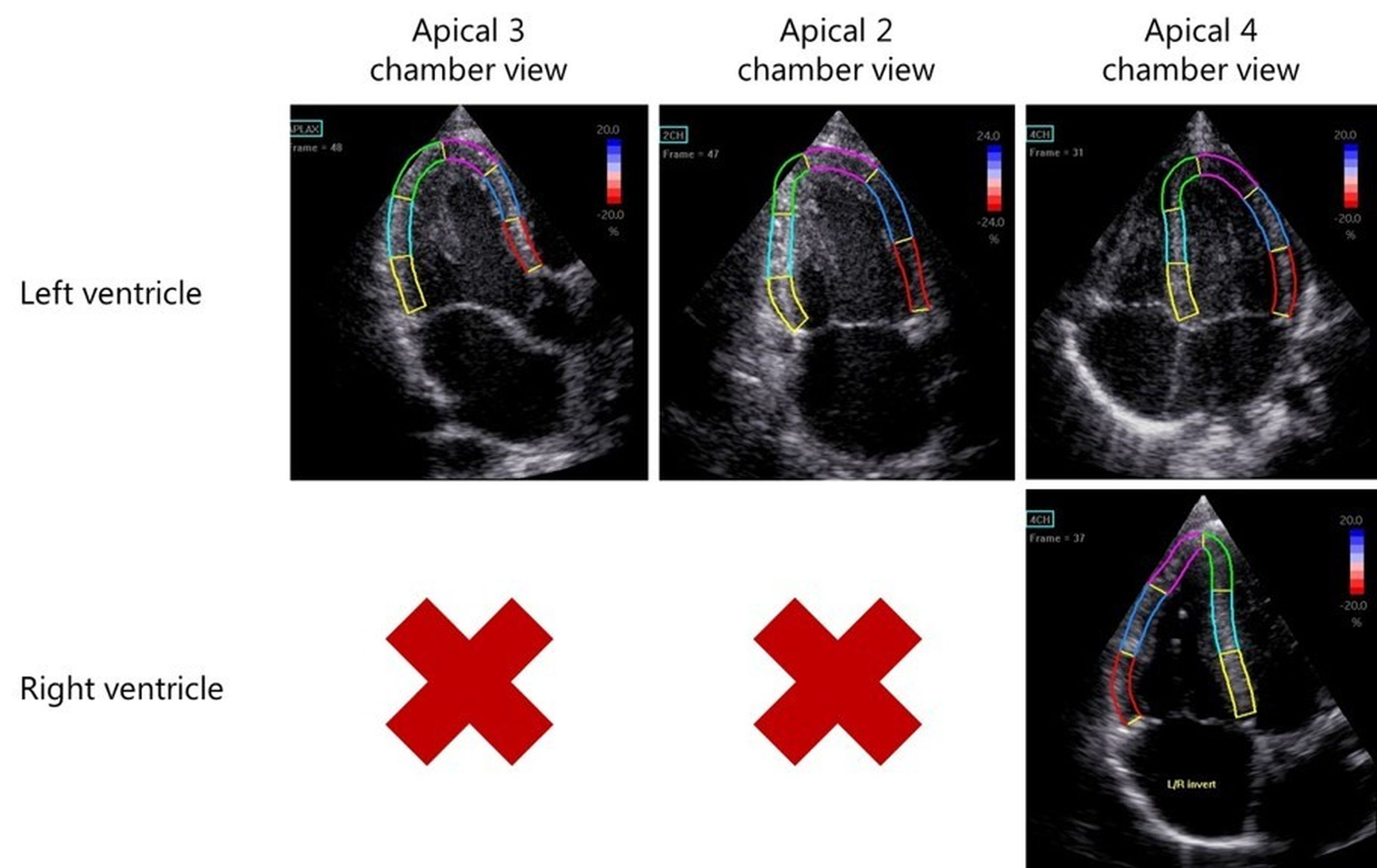

Supplement: Supplementary file 1 [file jcdd-12-00322-s001.zip › Supplemental Figure S3.tif]
